# Efficacy, Safety and Cost of Insecticide Treated Wall Lining, Insecticide Treated Bed Nets and Indoor Wall Wash with Lime for Visceral Leishmaniasis Vector Control in the Indian Sub-continent: A Multi-country Cluster Randomized Controlled Trial

**DOI:** 10.1371/journal.pntd.0004932

**Published:** 2016-08-17

**Authors:** Dinesh Mondal, Murari Lal Das, Vijay Kumar, M. Mamun Huda, Pradeep Das, Debashis Ghosh, Jyoti Priyanka, Greg Matlashewski, Axel Kroeger, Alexander Upfill-Brown, Rajib Chowdhury

**Affiliations:** 1 International Centre For Diarrhoeal Disease Research, Bangladesh (icddr,b), Shaheed Taj Uddin Ahmed Sarani, Mohakhali, Dhaka, Bangladesh; 2 BP Koirala Institute of Health Sciences, Entomology laboratory, Department of Microbiology, Dharan, Nepal; 3 Rajendra Memorial Research Institute of Medical Sciences, Patna, India; 4 Department of Microbiology and Immunology, McGill University, Montreal, Canada; 5 UNICEF/UNDP/World Bank/WHO Special Programme for Research and Training in Tropical Diseases (WHO/TDR), Geneva, Switzerland; 6 University Medical Centre Freiburg, Centre for Medicine and Society, Freiburg, Germany; 7 Center for World Health, David Geffen School of Medicine at UCLA, Los Angeles, California, United States of America; 8 National Institute of Preventive and Social Medicine, Mohakhali, Dhaka, Bangladesh; Liverpool School of Tropical Medicine, UNITED KINGDOM

## Abstract

**Background:**

We investigated the efficacy, safety and cost of lime wash of household walls plus treatment of sand fly breeding places with bleach (i.e. environmental management or EM), insecticide impregnated durable wall lining (DWL), and bed net impregnation with slow release insecticide (ITN) for sand fly control in the Indian sub-continent.

**Methods:**

This multi-country cluster randomized controlled trial had 24 clusters in each three sites with eight clusters per high, medium or low sand fly density stratum. Every cluster included 45–50 households. Five households from each cluster were randomly selected for entomological measurements including sand fly density and mortality at one, three, nine and twelve months post intervention. Household interviews were conducted for socioeconomic information and intervention acceptability assessment. Cost for each intervention was calculated. There was a control group without intervention.

**Findings:**

Sand fly mortality [mean and 95%CI] ranged from 84% (81%-87%) at one month to 74% (71%-78%) at 12 months for DWL, 75% (71%-79%) at one month to 49% (43%-55%) at twelve months for ITN, and 44% (34%-53%) at one month to 22% (14%-29%) at twelve months for EM. Adjusted intervention effect on sand fly density measured by incidence rate ratio ranged from 0.28 (0.23–0.34) at one month to 0.62 (0.51–0.75) at 12 months for DWL; 0.72 (0.62–0.85) at one month to 1.02 (0.86–1.22) at 12 months for ITN; and 0.89 (0.76–1.03) at one months to 1.49 (1.26–1.74) at 12 months for EM. Household acceptance of EM was 74% compared to 94% for both DWL and ITN. Operational cost per household in USD was about 5, 8, and 2 for EM, DWL and ITN, respectively. Minimal adverse reactions were reported for EM and ITN while 36% of households with DWL reported transient itching.

**Interpretation:**

DWL is the most effective, durable and acceptable control method followed by ITN. The Visceral Leishmaniasis (VL) Elimination Program in the Indian sub-continent should consider DWL and ITN for sand fly control in addition to IRS.

## Author Summary

Sand fly control is one of the main priorities of the Visceral Leishmaniasis (VL) Elimination Program in Bangladesh, India and Nepal. Currently, indoor residual spraying with insecticides (IRS) is the most effective method available for sand fly control. The efficacy of other methods of vector control remains poorly understood. To reach elimination, these additional techniques need to be explored in order to complement IRS or replace it if it is unavailable. In this multi-country study we investigated the efficacy, safety and cost of lime wash of household walls plus treatment of sand fly breeding places with bleach (EM), insecticide impregnated durable wall lining (DWL), and bed net impregnation with slow release insecticide (ITN) for sand fly control in the Indian sub-continent. Among these vector control methods, we found DWL to be the most effective tool for sand fly control followed by ITN. DWL and ITN should be considered, in addition to IRS, for sand fly control by the VL elimination program in the Indian sub-continent depending on need and affordability.

## Introduction

Visceral leishmaniasis (VL), also known as kala-azar, is a neglected vector-borne disease caused by the protozoan parasite *Leishmania donovani* and is transmitted by the female *Phlebotomus argentipes* sand fly. The poorest of the rural areas of Bangladesh, India, Nepal and East Africa are the victims of the disease which is fatal if not treated [1]. Approximately 200,000–400,000 cases of VL occur each year worldwide with a case fatality of 10% [1]. Bangladesh, India and Nepal contribute up to 60% of the VL burden in the world [2]. The Governments of Bangladesh, Indian and Nepal committed to eliminate VL as a public health problem with a target of less than 1 case per 10,000 people at upazila, district and block level in Bangladesh, Nepal and India respectively by 2017 [2,3].

Integrated vector management (IVM) is one of the strategies of the VL elimination program and is mostly depending on indoor residual spraying with insecticides (IRS). Insecticide treated bed nets (ITNs), and environmental management (EM) (lime wash of household walls with mud or lime, and treatment of breeding sites with chemicals or insecticide) can complement IRS in IVM. However, there are conflicting results regarding the efficacy of ITNs and EM. A 24% reduction in sand fly density by the use of ITN has been reported in India and Nepal, whereas, Mondal et al observed a 65% reduction in sand fly density through the ITN program in Bangladesh [4,5]. Similarly, EM was found to be effective in Nepal but not in India or Bangladesh [6]. It is difficult to directly compare these results because of differences in study designs and intervention applications.

In addition, there are newly developed vector control tools that have yet to be evaluated. Insecticide treated Durable Wall Lining (DWL, ZeroVector^™^, Vestergaard, Switzerland) contains a thin polyethylene material impregnated with deltamethrin. It has been found to be effective against malaria transmitting mosquitoes in Africa [7], but its efficacy and toxicity to sand flies have never been tested before.

We therefore undertook a single design study in all three member countries of the elimination initiative to investigate the efficacy, duration, cost and safety of environment friendly vector control interventions (ITNs and EM) and also DWL [7].

In this project we aimed to compare the efficacy, safety and cost of DWL, ITN and EM in houses of VL endemic areas in Bangladesh, India and Nepal (ClinicalTrials.gov Identifier: NCT01644682).

## Methods

### Study area and population

The study was a multi-centre cluster randomized controlled trial comparing three vector control methods in VL endemic villages of Bangladesh, India and Nepal from November, 2011 to November 2013.

### Study design and sampling

#### Selection of the study villages

**Fig 1:**
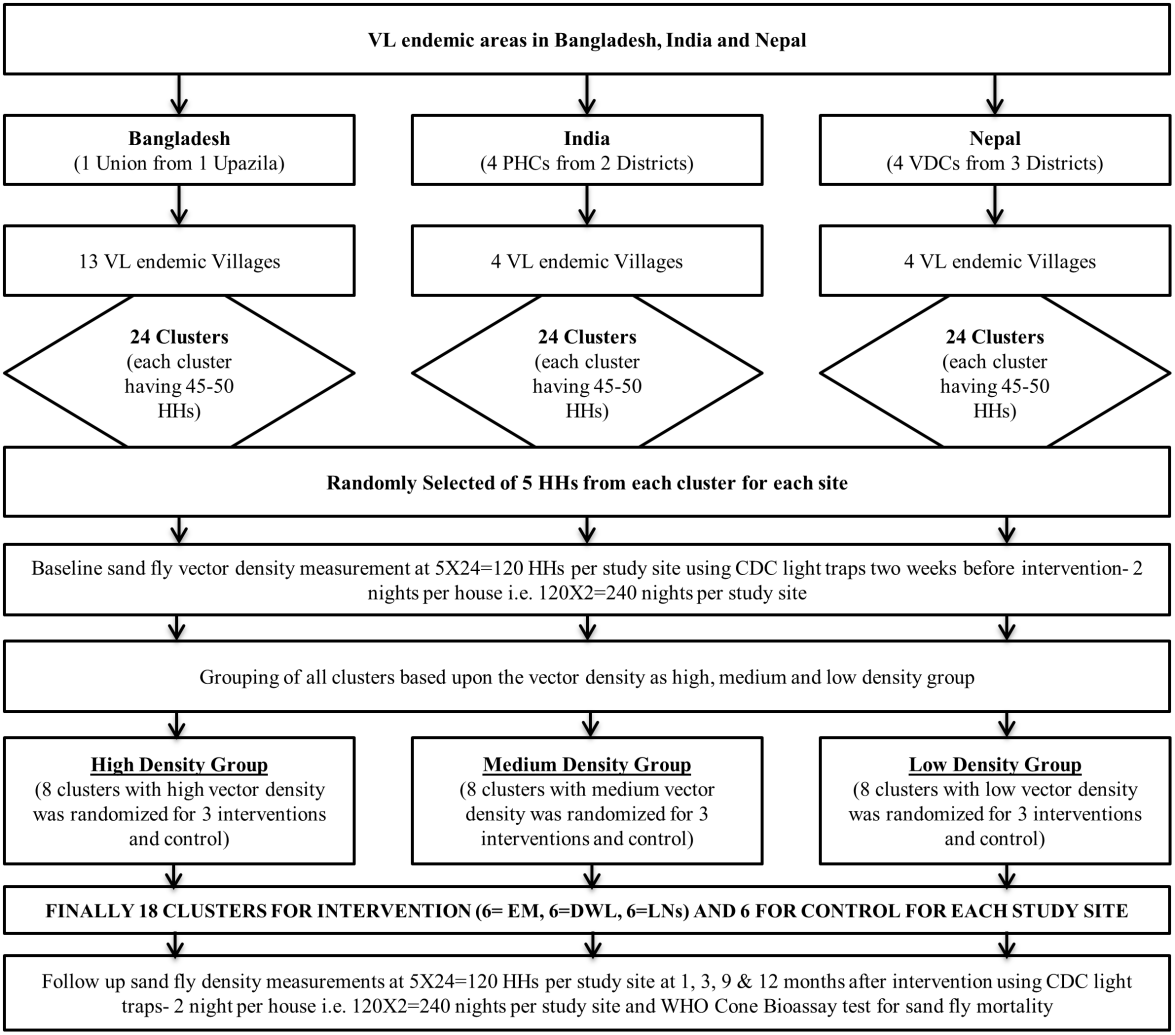
Study design.

Each country team collected information from government health authorities about reported VL cases by villages from the upazila health complex, district hospital and primary health centre respectively in Bangladesh, Nepal and India. The selected villages had VL cases in the last three years. A total of 21 (13, 4 and 4 from Bangladesh, India and Nepal respectively) VL endemic villages were selected and details of the sampling procedures are presented in Fig 1.

#### Selecting clusters of households within villages

The study villages were sub-divided into smaller units containing 45–50 households (HHs) which represented a cluster. A minimum of 50 meters distance between two clusters was maintained to avoid contamination of the intervention. The total number of clusters in each study site was 24.

#### Selection of HHs for entomological activities

Five out of 50 HHs from each cluster were selected randomly for measuring the sand fly density at 2 weeks before intervention (baseline) and at 1, 3, 9 and 12 months after intervention. The WHO Cone bioassay test for assessment of sand fly mortality after exposure of sand flies to treated nets, DWL surfaces and lime washed walls was performed at 1, 3, 9 and 12 months after interventions.

### Allocation of clusters for intervention and control

The study clusters in a particular site were stratified based on baseline sand fly distribution into low density (bottom third) medium density (middle third) and high density (top third) groups. Of the 24 clusters per site, 8 clusters were therefore allocated to each low, middle and high density group. These 8 clusters in each group were randomized with 2 clusters received EM; 2 receiving DWL, 2 receiving ITN; and 2 with no intervention as the control. Randomization was performed using Random Allocation Software, version: 1.0.0 [8].

### Interventions and their implementation

#### Intervention 1(Environmental Management, EM)

EM included indoor household wall washed with lime and treatment of sand fly breeding places (cattle shed and places around household) with bleach. The Regional Technical Advisory Group for VL elimination program in the Indian sub-continent suggested EM as a VL vector control method [2].Several studies in the past established bleach as an effective mosquito ovicide / larvicide substance [9–11]. Kumar et al found mud and lime plaster as an effective tool for sand fly control in India [12]. We, therefore, chose lime wash and bleach as EM tool for sand fly control. We used usual household bleach powder which had 35% active chlorine concentration. We educated community volunteers about safety during deploying lime and bleach especially to avoid any direct contact and inhalation with these chemicals, mandatory use of personal protective equipments (PPE), and to avoid mixing of materials, eating and smoking during intervention. Trained research assistants meticulously monitored proper implementation of the interventions and compliance of community volunteers with safety instructions.

#### Intervention 2 (Durable Wall Lining, DWL)

DWL included installation of commercial deltamethrin impregnated durable wall lining. As per manufacturer specification the concentration of deltamethrin in DWL is 170 mg a.i. / m^2^. Trained village volunteers installed DWL in the allocated households under supervision of trained field research supervisors. External experts monitored and approved the proper installing of DWL in the study households.

#### Intervention 3 (Impregnated Bed Nets, ITN)

ITN included impregnation of bed nets with slow release insecticide tablets KO Tab 123 (Bayer Ltd., Isando, South Africa). This converts a bed net into a long-lasting insecticide treated net (LLIN). Entomological evaluation found that bed-nets impregnated with KO Tab 123 were as effective as PermaNet 2.0 [13]. Trained community volunteers impregnated the nets under supervision of the research team. Bed nets were dipped in a freshly prepared solution of KO Tab 123 containing 0.4 g deltamethrin in a 1.6 g tablet and a chemical binder according to manufacturer instructions. In this way a concentration of about 25mg a.i. / m^2^ of net can be achieved [5]. Most of the nets were of polyester materials and were “family size” (usually 2.3 m x 1.8 m in size and mess size between 1.2–1.5mm). The research team monitored the HH use of ITNs by direct observation between 9.00 to 10.00 pm in every 10^th^ HHs.

All interventions had been conducted once only and all households in a cluster received the allocated intervention. We did not repeat EM, DWL and ITN intervention during follow up. We monitored physical status of DWL, ITN and lime washed walls at six weeks after intervention in addition to monitoring impregnated bed net use as mentioned above.

#### Control arm

Control HHs received no intervention and received a commercially impregnated bed net after the study was completed.

### Household head interview

Information related to the HHs socio-economic status, knowledge and practice surrounding VL and ownership of bed nets was collected through interviewing HH heads in clusters with and without intervention using a structured questionnaire.

### Measurement of efficacy of interventions

The efficacy of each intervention was assessed by sand fly mortality and indoor sand fly density reduction (see below). Entomological activities were done in the same HHs of a cluster where baseline sand fly density measurement was carried out.

#### Sand fly density measurement

Sand flies were collected on 2 consecutive nights from 6.00 pm to 6.00 am using a CDC light trap at baseline (two weeks before intervention) and then at 1, 3, 9 and 12 months after intervention as described previously [5]. The sand fly density was expressed by number of female *Phlebotomus argentipes* count per HH. Sand fly species identification was performed by morphological examination [14].

#### Sand fly mortality assessment

A WHO cone method (WHOPES 2005.11) was conducted on treated materials or surface using manually collected sand flies [15]. We did not determine baseline susceptibility of wild sand fly population to deltamethrin, because studies prior to our study in VL endemic areas of the three countries demonstrated that wild sand fly population was highly susceptible to deltamethrin [16–17]. Bioassays were performed at 25°- 29°C and 75%-85% humidity. Sand flies (10–12 female) were introduced in the cones and placed on 5 surfaces (four sides and top) of the net, and four surfaces of DWL and lime washed walls. Sand flies were exposed for 3 minutes to DWL and ITN and for 30 minutes against lime washed wall. The sand flies were then transferred to a paper cup and observed for 24 hours to determine mortality. Control cones were placed on non-treated surfaces. The mortality rates of sand flies were corrected using Abbot’s formula (WHOPES 2005.11) [15].

### Acceptability survey for sand-fly control intervention

Trained field research assistants interviewed HH heads using a structured questionnaire at 6 weeks after the intervention to collect data on adverse events, insect nuisances and other concern about the specific intervention in his/her HH.

### Estimation of operational cost per intervention

Data included costs related to preparatory and monitoring activities, quantity of materials and associated unit costs, and operational cost related to intervention implementation (community volunteers’ fee, travel and expenses for accessories). Average material cost per household covered expenses of quantity material used per household. Similarly, average operational cost comprised average expenses of implementation for each intervention per household.

### Sample size calculation

The sample size estimation was based on the vector densities and distributions documented in previous entomological studies and sand fly reduction rates in similar intervention studies performed in Venezuela, Bangladesh, India and Nepal [6, 18]. We assumed that the distribution of sand fly counts would follow a negative binomial distribution with a dispersion coefficient of *k* = 0.05 and an intra cluster coefficient of 0.03, a reduction from 20 to 5 vectors per trap / per night, and an average of 50 households per cluster. The minimum sample size was found to be 6 clusters per intervention, with a total of 24 clusters per study site to achieve 80% power and a significance level of 5%. The study was individually powered for each study site.

### Quality control

The study was conducted as per good clinical practice. In addition to the internal monitoring of study activities by investigators, the study was monitored by an international monitor and experts.

### Statistical analysis

The three study sites collected data using standardized data collection tools with error checking and correction done via comparison against field data. Data from the all sites were sent to icddr,b in Bangladesh where they were merged in one data base, checked for consistency and duplicates, and corrected before analysis. The analysis was done using univariate, bivariate and multivariate techniques where applicable. Main outcome variables were sand fly mortality and reduction in indoor sand fly density. Female *P*. *argentipes* sand fly counts before and after the intervention were compared using a non-parametric approach (Mann Whitney U test). The crude intervention effect was then estimated as the difference in differences and should be zero if there was no intervention effect and negative if there was a reduction in the intervention groups compared to the control group. Effect of intervention on sand fly count at household level was calculated as (B-A)-(D-C): A = baseline value for the intervention group; B = follow-up value for the intervention group; C = baseline value for the control group; D = follow-up value for the control group. The average effect size with 95% CI at household level was calculated.

Female *P*. *argentipes* counts were assumed to fit a Poisson distribution. Generalized estimating equation (GEE) models were used to adjust dependency in observations due to repeated measurement at different follow-up times in cluster sampling design. The regression model had the following structure: Count = Intercept + a*Treatment + b*Time + c*Interaction + error, where treatment was 1 for intervention and 0 for control; time was 0 for baseline and 1 for follow up, and interaction was 1 for intervention group at follow up. In the model “c” represents intervention effect which was reported in exponential form as incidence rate ratio (IRR). Variables related to socio-economic status, knowledge about VL and its vector, and protection from sand fly and mosquito bites were dichotomized before analysis. Household asset score was generated by principal component analysis using the following variables: electricity, radio, television, mattress, bed net, motor cycle, bicycle, van, power tiller, shallow machine, chair/table, mobile phone, clock, sewing machine, and fishery. We trisected the score into equally sized low, medium and high asset groups. The variables associated with interventions with P-values ≤ 0.20 were considered as covariates in the regression model. P-values were considered statistically significant at a level of 0.05 and 95% confidence intervals were reported for all estimates where applicable. All the analysis was perform by using STATA 10.1.

### Ethical consideration

The ethical review committees of the International Centre For Diarrhoeal Disease Research, Bangladesh (icddr,b), the Rajendra Memorial Research Institute of Medical Sciences (RMRIMS) and the B.P. Koirala Institute of Health Sciences (BPKIHS) respectively in Bangladesh, India and Nepal approved the study. Written informed voluntary consent was obtained from the HH heads before conducting any study related activities.

## Results

### Population characteristics concerning VL and the vector

Table 1 presents the number of districts, sub-districts, villages and clusters, included in the study. The total number of study HHs was 3667 with 16861 inhabitants with similar numbers across intervention clusters. Study participants had high levels of illiteracy (59%) and many were unskilled laborers (50%). The families were large (50% with >4 members) and lived mostly in one bed room houses (52%). The houses were mostly built of mud (67%), had a mud floor (95%) and had cracks in their walls (79%). The household asset score was mainly low or medium (64%). There were only minor differences between intervention and control clusters regarding socio-economic indicators (Table 2). However, intervention clusters differed from the control clusters with respect to HHs head’s knowledge about VL, bed-net ownership and bed-net use (Table 2).

**Table 1:** Study profile: Study clusters, households and population by interventions in Bangladesh, India and Nepal.

	Bangladesh	India	Nepal	Pooled
**Number of district**	1	2	2	5
**Number of Upazila/Primary Health Centre (PHC)/Village Development Committee (VDC)**	1	4	4	9
**Number of village**	13	4	4	21
**Number of cluster**	24	24	24	72
EM	6	6	6	18
DWL	6	6	6	18
ITN	6	6	6	18
Control	6	6	6	18
**Total household**	1184	1199	1284	3667
EM	297	299	305	901
DWL	296	300	327	923
ITN	291	300	349	940
Control	300	300	303	903
**Total population**	5103	5899	5859	16861
EM	1319	1470	1365	4154
DWL	1203	1465	1602	4270
ITN	1339	1458	1545	4342
Control	1242	1506	1347	4095

EM: Environmental management; DWL: Durable wall lining; ITN: Impregnation of bed nets with KO Tab 123; Control: no intervention

**Table 2:** Socio-demographic characteristics, knowledge about VL and vector control practice in intervention versus control clusters (Pooled data).

	Control Arm, % (n)	Intervention Arms						Total, % (n)
		EM^a^, % (n)	P-value	DWL^b^, % (n)	P-value	ITN^c^, % (n)	P-value	
	N = 90	N = 90		N = 90		N = 90		N = 360
**Illiterate household head**	55.6 (50)	71.1 (64)	0.030	57.8 (52)	0.764	52.2 (47)	0.654	59.2 (213)
**Unskilled household head**	54.4 (49)	51.1 (46)	0.654	58.9 (53)	0.547	35.6 (32)	0.011	50.0 (180)
**Family size > 4**	51.1 (46)	43.3 (39)	0.296	51.1 (46)	1.000	54.4 (49)	0.654	50.0 (180)
**Bed-rooms <2**	54.4 (49)	54.4 (49)	1.000	55.6 (50)	0.881	42.2 (38)	0.101	51.7 (186)
**Family members slept at Varanda during the hot season**	62.2 (56)	72.2 (65)	0.153	63.3 (57)	0.877	57.8 (52)	0.543	63.9 (230)
**Having cattle shed**	37.8 (34)	48.9 (44)	0.133	53.3 (48)	0.036	51.1 (46)	0.072	47.8 (172)
**Housing materials:**								
Mud wall	63.3 (57)	74.4 (67)	0.107	66.7 (60)	0.639	62.2 (56)	0.877	66.7 (240)
Mud floor	95.6 (86)	97.8 (88)	0.682	96.7 (87)	1.000	91.1 (82)	0.232	95.3 (343)
**HH asset score:**								
Low	32.2(29)	27.8(25)	0.808	32.2(29)	0.935	36.7(33)	0.045	32.2(116)
Medium	27.8(25)	30.0(27)		30.0(27)		40.0(36)		31.9(115)
High	40.0(36)	42.2(38)		37.8(34)		23.3(21)		35.8(129)
**Cracks in household indoor wall**	73.3 (66)	90.0 (81)	0.004	82.2 (74)	0.151	72.2 (65)	0.867	79.4 (286)
**Damp floor**	4.4 (4)	4.4 (4)	1.000	5.6 (5)	1.000	15.6 (14)	0.013	7.5 (27)
**HH head aware about VL**	85.6(77)	94.4(85)	0.047	90.0(81)	0.363	81.1(73)	0.424	87.8(316)
**HH head aware about VL vector**	20.0(18)	18.9(17)	0.851	10.0(9)	0.060	4.4(4)	0.001	13.3(48)
**Having bed-net in house**	75.6(68)	73.3(66)	0.733	74.4(67)	0.863	100.0(90)	0.000	80.8(291)
**Regular use of bed-net**	30.0(27)	38.9(35)	0.210	45.6(41)	0.031	45.6(41)	0.031	40.0(144)
**Other insecticides use for mosquito control:**								
Mosquito coil	10.0(9)	8.9(8)	0.799	7.8(7)	0.600	12.2(11)	0.635	9.7(35)
Repellents	0.0(00)	3.3(3)	0.246	0.0(00)	--	0.0(00)	--	0.8(3)
Spray	0.0(00)	1.1(1)	1.000	0.0(00)	--	0.0(00)	--	0.3(1)
Smoke/dhup	27.8(25)	27.8(25)	1.000	26.7(24)	0.867	28.9(26)	0.869	27.8(100)
Others	1.1(1)	1.1(1)	1.000	0.0(0)	1.000	2.2(2)	1.000	1.1(4)
**House sprayed with insecticide (IRS) in the last 6 months**	36.7(33)	45.6(41)	0.226	38.9(35)	0.758	36.7(33)	1.000	39.4(142)

^a^EM: Environmental management;

^b^DWL: Durable wall lining;

^c^ITN: Impregnation of bed nets with KO Tab 123

### Efficacy of interventions

Efficacy of interventions was assayed by assessment their effects on sand fly mortality and on reduction of sand fly density.

#### Effect on sand fly mortality

**Fig 2:**
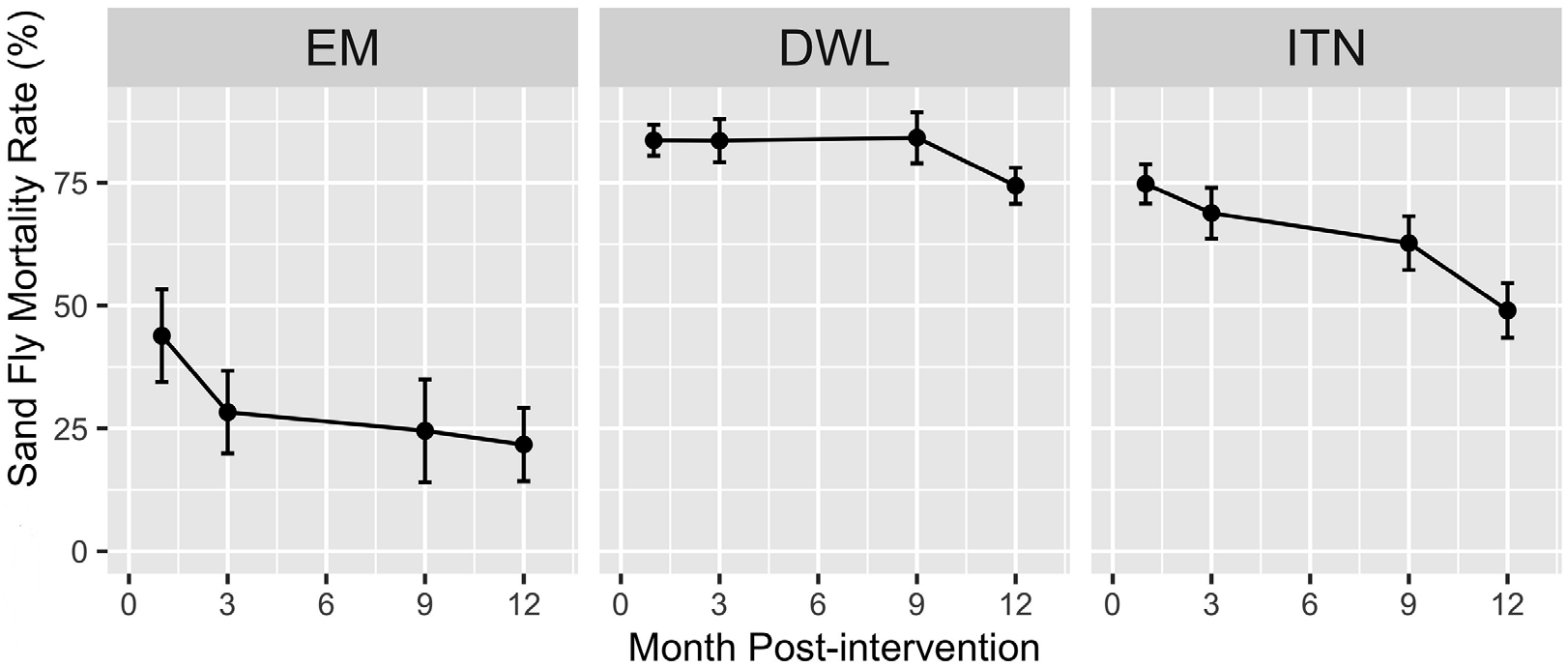
Abbot-corrected *P*. *argentipes* sand fly mortality by intervention at follow up periods. Error bars represent 95% CI.

The Abbott’s corrected sand fly mortality rate [mean and 95%CI] at 1, 3, 9 and 12 months after interventions was respectively 84% (81%-87%), 84% (79%-88%), 84% (79%-89%) and 74% (71%-78%) for DWL, 75% (71%-79%), 67% (64%-74%), 63% (57%-68%) and 49% (43%-55%) for ITN, and 44% (34%-53%) 28% (20%-37%), 25% (14%-35%), 22% (14%-29%) for EM (Table 3). The effect on sand fly mortality of DWL remained superior to ITN and EM, and that of ITN to EM during follow up (Fig 2).

**Table 3:** Abbot-corrected *P*. *argetipes* sand fly mortality by interventions and follow up.

	Average corrected sand fly mortality (95% CI)		
Time	EM^a^	DWL^b^	ITN^c^
**At 1-month follow-up**	43.87% (34.43%–53.32%)	83.65% (80.51%–86.79%)	74.77% (70.75%–78.79%)
**At 3-month follow-up**	28.32% (19.90%–36.74%)	83.57% (79.22%–87.93%)	68.84% (63.65%–74.02%)
**At 9-month follow-up**	24.51% (14.04%–34.98%)	84.17% (78.97%–89.36%)	62.73% (57.26%–68.19%)
**At 12-month follow-up**	21.72% (14.24%–29.19%)	74.39% (70.71%–78.07%)	49.02% (43.45%–54.59%)

^a^EM: Environmental management;

^b^DWL: Durable wall lining;

^c^ITN: Impregnation of bed nets with KO Tab 123

#### Effect of sand fly density

At baseline, sand fly density (female *Phlebotomus argentipes*) was similar in intervention and control HHs (Table 3). At one month after the intervention, the sand fly density was less in HHs with EM compared to control households, but the effect was very short lived (Table 4). In the DWL clusters, sand fly densities were lower over the entire 12-months period compared with control clusters. In the ITN clusters the sand fly density was also reduced, however the effect only lasted through 9 months.

**Table 4:** Female *Phlebotomus argentipes* sand fly per household and their comparison between intervention versus control cluster at baseline and follow-up.

	*Female P*. *argentipes sand fly per household; Mean (95% CI)* [P-value*]				Average effect on count per *household;* [95% CI] ^ǂ^		
Time	EM^a^	DWL^b^	ITN^c^	Control^d^	EM^a^ vs. Control^d^	DWL^b^ vs. Control^d^	ITN^c^ vs. Control^d^
**Baseline**	5.4 (4.11, 6.69)[0.879]	5.48 (4.13, 6.82)[0.621]	4.60 (3.79, 5.41)[0.528]	5.92 (4.40, 7.44)	--	--	--
**1-month follow-up**	7.62 (5.34, 9.91)[0.018]	2.44 (1.25, 3.64)[<0.0001]	5.27 (3.62, 6.91)[<0.0001]	9.39 (7.63, 11.14)	-1.24 [-3.25, 0.76]	-3.28[-4.61, -1.95]	-2.8[-4.82, -0.77]
**3-month follow-up**	5.96 (3.82, 8.09)[0.385]	1.23 (0.86, 1.61)[0.001]	2.96 (2.15, 3.77)[0.949]	4.96 (3.17, 6.74)	1.52[0.75, 2.29]	-3.06[-5.62, -0.5]	-0.68[-1.35, -0.006]
**9-month follow-up**	5.56 (3.85, 7.26)[0.181]	1.92 (0.87, 2.97)[<0.0001]	2.30 (1.67, 2.93)[0.024]	5.42 (1.69, 9.16)	0.66[-2.48, 3.79]	-1.33[-2.81, 0.14]	-1.8[-4.46, 0.86]
**12-month follow-up**	5.64 (3.50, 7.79)[0.025]	2.39 (1.00, 3.78)[0.005]	3.38 (2.08, 4.68)[0.279]	4.17 (1.25, 7.08)	2.0[0.18, 3.82]	-2.8[-4.82, -0.78]	0.53[-1.71, 2.78]
**Average (1–12) months follow-up**	6.20 (4.13, 8.26)	2.00 (1.00–3.00)	3.48 (2.38, 4.57)	6.00 (3.44, 8.53)	0.74[-1.2, 2.67]	-2.62[-4.47, -0.77]	-1.19[-3.09, 0.72]

^a^EM: Environmental management;

^b^DWL: Durable wall lining;

^c^ITN: Impregnation of bed nets with KO Tab 123,

^**d**^Control = no intervention

*P-value for test of mean differences between intervention and control arms

^ǂ^ Crude estimated effect in Female *P*. *argentipes* sand fly counts attributed by the intervention compared to control arm. Please see the calculation in statistical analysis.

When adjusted for confounders in regression analysis, DWL remained the most efficacious intervention for reducing sand fly density followed by ITN (Table 5). The efficacy of DWL was significant for 12 months and that of ITN was significant for 9 months. The EM was not found to have significant effect on sand fly reduction by regression analysis (Table 5).

**Table 5:** Effect of intervention on female *P*. *argentipes* densities adjusted for covariates by longitudinal regression analysis.

Time/Model	Parameter	IRR [95% CI](P-value)		
		EM^a^	DWL^b^	ITN^c^
**1-month follow-up**				
Simple model	Crude Intervention effect*	0.89 [0.76, 1.03] (0.122)	0.28 [0.23, 0.34] (<0.0001)	0.72 [0.62, 0.85] (<0.0001)
Full model	Adjusted Intervention effect	0.89 [0.76, 1.03] (0.113)^$1^	0.28 [0.24, 0.34] (<0.0001)^$2^	0.72 [0.62, 0.85] (<0.0001)^$3^
**3-month follow-up**				
Simple model	Crude Intervention effect*	1.32 [1.13, 1.54] (<0.0001)	0.27 [0.21, 0.34] (<0.0001)	0.77 [0.64, 0.91] (0.003)
Full model	Adjusted Intervention effect	1.32 [1.13, 1.54] (<0.0001)^$1^	0.27 [0.21, 0.34] (<0.0001)^$2^	0.77 [0.64, 0.91] (0.003)^$3^
**9-month follow-up**				
Simple model	Crude Intervention effect*	1.12 [0.96, 1.32] (0.156)	0.38 [0.31, 0.47] (<0.0001)	0.55 [0.45, 0.66] (<0.0001)
Full model	Adjusted Intervention effect	1.12 [0.96, 1.32] (0.160)^$1^	0.38 [0.31, 0.47] (<0.0001) ^$2^	0.53 [0.44, 0.65] (<0.0001)^$3^
**12-month follow-up**				
Simple model	Crude Intervention effect*	1.49 [1.26, 1.74] (<0.0001)	0.62 [0.51, 0.75] (<0.0001)	1.04 [0.88, 1.24] (0.629)
Full model	Adjusted Intervention effect	1.49 [1.26, 1.74] (<0.0001)^$1^	0.62 [0.51, 0.75] (<0.0001) ^$2^	1.02 [0.86, 1.22] (0.792)^$3^

^a^EM: Environmental management;

^b^DWL: Durable wall lining;

^c^ITN: Impregnation of bed nets with KO Tab 123

*The intervention effect and covariates are tested in two types of longitudinal regression models (GEE with Poisson distribution model) at four different follow-up times; simple not controlling for any covariates, full model controlling covariates. The variables which varied between intervention and control areas with P-values less than equal 0.20 are considered as covariates for full model. IRR with 95% CI and P-values for the regression parameter of intervention effect are presented only. Regression analysis was performed by considering clustering affect.

^$1^Full model adjusted by the covariates: Illiterate HH head, Slept at Varanda, Having cattle shed, Mud wall, Crack in wall, HH aware about VL

^$2^Full model adjusted by the covariates: Having cattle shed, Crack in wall, HH aware about VL vector, Regular use of bed-net

^$3^Full model adjusted by the covariates: Labor HH head, Bed-room < 2, Having cattle shed, HH asset score, Damp floor, HH head aware about VL vector, Having bed-net, bed-net <2 in house, Regular use of bed-net

### Acceptability and safety

About 94% of the 891 interviewees liked DWL and ITN (Table 6). The acceptance rate was much lower for EM (79%). Most of the HHs maintained DWL intact and very few of the HH with ITN had washed their bed nets (Table 6) which indicate a very good acceptability of these interventions. In general interventions had low levels of adverse events with the exception of DWL, where itching was reported in 36% of the interviewees (almost exclusively in India) which was transient in nature and did not need any medication. Transient itching was also reported by 4.8% of HHs with ITN. Unpleasant smell was reported in 3.2% of ITN houses (though the insecticide is odorless). Other adverse effects such as coughing / dizziness were very rare (0%-0.5%) (Table 6).

**Table 6:** Safety and acceptability of interventions.

	Intervention		
Indicator	EM^a^	DWL^b^	ITN^c^
Itching, % (n/N) [95% CI]	0.0 (0/878) [--]	36.0 (321/891) [32.9, 39.28]	4.8 (40/838) [3.43, 6.44]
Unpleasant smell, % (n/N)[95% CI]	1.1 (10/878) [0.5, 2.08]	0.6 (5/891) [0.18, 1.30]	3.2 (27/838) [2.13, 4.65]
Cough, dizziness, % (n/N)[95% CI]	0.0 (0/878) [--]	0.3 (3/891) [0.07, 1.0]	0.5 (4/838) [0.13,1.22]
**Acceptability**			
Like the intervention, % (n/N)[95% CI]	78.5 (689/878) [75.60, 81.15]	93.7 (835/891) [91.92, 95.22]	93.9 (787/838) [92.10,95.44]
Plastered HH wall with mud after EM intervention, % (n/N)[95% CI]	3.8 (33/878) [2.60, 5.24]	NA	NA
Physically intact DWL, % (n/N)[95% CI]	NA	97.5 (869/891) [96.29, 98.45]	NA
Average frequency of treated bed-net(s) washed [95% CI]	NA	NA	0.21 [0.14–0.29]

^a^EM: Environmental management;

^b^DWL: Durable wall lining;

^c^ITN: Impregnation of bed nets with KO Tab 123; NA = not applicable

Average operational cost per HH for EM, DWL and ITN was respectively USD$4.83, $7.57 and $1.47 (Table 7). The average operational cost per household was more for DWL and EM compared to their materials cost per HH (Table 7). The average combined cost per HH was highest for DWL ($13.57) followed by EM ($5.19) and ITN ($3.62)

**Table 7:** Material and operational cost* by intervention and study site.

Study Site	Bangladesh	India	Nepal	All sites’ average
Intervention**	**Number of households**			
EM	297	299	305	300
DWL	296	300	327	308
ITN	291	300	349	313
Total	884	899	981	921
**Activity Type**	**Preparatory and Monitoring Activity cost**			
Meeting and training	285	1253	439	659
Trainer, field staff (N)	12039 (4)	9555 (4)	3321 (3)	8305 (3.3)
Monitoring (# visit)	407 (2)	81 (1)	244 (3)	244 (2)
Total cost	12731	10889	4004	9208
**Cost per household**	14.40	12.11	4.08	10.2
**Intervention**	**Total quantity of materials and their unit cost**			
**EM**				
Lime in Kg (cost per Kg)	1470 (0.15)	1500 (0.24)	650 (0.17)	1206.67 (0.17)
Bleach powder in Kg (cost per Kg)	240 (0.68)	300 (0.36)	100 (0.51)	213.33 (0.51)
**DWL**				
Number of Roll (cost per roll)	37 (50)	32 (50)	45 (50)	38 (50)
**ITN**				
number of KO Tab123 (cost per tablet)	692 (1.0)	462 (1.0)	883 (1.0)	679 (1.0)
**Intervention**	**Average quantity of materials and cost per household**			
**EM**				
Quantity of lime in Kg	4.95	5.01	2.13	4.03
Quantity of bleach in Kg	0.81	1.0	0.33	0.71
Cost	0.55	0.36	0.17	0.36
**DWL**				
Quantity of roll	0.13	0.11	0.14	0.13
Cost	6.50	5.50	7.00	6.00
**ITN**				
Tablets in number	2.38	1.54	2.53	2.15
Cost	2.38	1.54	2.53	2.15
**Intervention**	Total and per household (HH) operational cost***			
**EM**				
Total cost (per HH cost)	888 (2.99)	1901(6.36)	1568 (5.14)	1452.33 (4.83)
**DWL**				
Total cost (per HH cost)	1701(5.75)	2419 (8.06)	2913 (8.91)	2344.33 (7.57)
**ITN**				
Total cost (per HH cost)	124 (0.43)	411(1.37)	909 (2.60)	481.33 (1.47)

*All cost in USD;

** EM: Environmental management; DWL: Durable wall lining; ITN: Impregnation of bed nets with KO Tab 123;

***Excluding intervention materials cost like Lime, Bleach powder, DWL and KO Tab 123. This includes personnel, travel and accessories cost. Accessories includes Bucket, Mug, Soap, Towel, Gloves, Brush for painting, Dram for EM; Hammer, Nails, Scissors, Gloves, Pliers, Torch light, Measuring tap, Plastic such as trash bag, Soap, Washer, Towel for DWL; and Bucket, Mug, Soap, Towel for ITN.

## Discussion

The VL Elimination Program in the Indian sub-continent recommends sand fly reduction through integrated vector control management (IVM) which optimally requires more than one vector control method [2]. IRS is widely used by the program but other vector control methods like EM, ITN and DWL are not. To date, only two RCTs in the Indian sub-continent for sand fly control had been carried out, and one of these two studies included sites in all three countries [4,6]. This study is the only RCT in this region where a common protocol was followed across all sites in the study of EM and ITN. Furthermore, the current study is the first to examine the effect of DWL on sand fly mortality and density reduction.

We found DWL to be the most effective and long-lasting method for controlling the female *Phlebotomus argentipes*. The costs of DWL are relatively high for a public health program, however. Further studies are needed to determine whether reducing wall coverage (up to a height of 150 cm or less) remains sufficient for controlling sand flies. Such a study would be warranted as sand flies move by hopping slowly upwards and usually remain on the lower parts of the wall. The high proportion of itching in DWL households, concentrated mostly in India will require further analysis.

The effectiveness of bed net impregnation for sand fly control has been described previously in Bangladesh [5]. Ours is the first study where usefulness of bed net impregnation with slow release insecticide tablets in sand fly control has been evaluated and confirmed in India and Nepal. The duration of efficacy (9 months) is less in this study compared to the duration (18 months) reported previously in Bangladesh [5]. This may be explained by operational errors during bed net impregnation and the breakdown of insecticide in nets over time [5]. Commercial LLINs are advantageous in this regard as they are less susceptible to operational errors and rapid insecticide loss. The unit price of manually impregnated ITNs, however, is less than that of the commercial LLINs. A well designed cost-effectiveness study for sand fly control with bed-net impregnation with slow release insecticide tablet versus commercial LLIN will be useful to guide the policymaker for selection of better method for sand fly control.

Bed net impregnation with community involvement as shown in this study was affordable and operationally feasible in a public health program where village health workers played an important role. The EM approach used in this study including treatment of outside sand fly breeding sites was not effective in reducing sand fly density, and the associated sand fly mortality was lowest of the three interventions. The operation cost was also substantially higher than that of ITN though the material cost was lower. These findings are consistent with a previous study where EM effectiveness was not robust [6]. Therefore, we do not recommend indoor household wall lime wash or deploying bleach in suspected sand fly breeding places for sand fly control.

The major limitation of this study is the inability to use an epidemiological endpoint due to low incidence of VL. However, in order to expect such an effect demonstration of reduced sand fly density and increase mortality is necessary and that was the aim here. Information regarding effectiveness, safety, and cost of these interventions is sufficient to demonstrate their usefulness as alternative vector control methods for the VL Elimination Program. Larger-scale studies following widespread role out of these interventions would be able to evaluate their effect of VL incidence.

In conclusion, ITN and DWL were found to be efficacious for controlling sand fly in the Indian sub-continent. The major advantage of ITNs is their lower price while their limitation is the shorter duration of efficacy compared to the DWL. On the other hand, the advantage of the DWL is the higher efficacy with a longer duration of action while the major disadvantage is the higher cost. We hope that this study results will be useful to policy makers in the selection of sand fly control methods in a situation when the number of VL cases is reduced in all three countries [19, 20].

## References

[1] Alvar J, Vélez ID, Bern C et al Leishmaniasis Worldwide and Global Estimates of Its Incidence. PLoS ONE 2012; 7: e35671 10.1371/journal.pone.0035671 22693548 PMC3365071

[2] Regional Strategic Framework for Elimination of Kala-azar from South-East Asia Region (2005–2015). WHO (SEARO) 2005.

[3] Mondal D, Alvar J, Hasnain MG et al Efficacy and safety of single dose liposomal amphotericin B for visceral leishmaniasis in a rural public hospital in Bangladesh: a feasibility study. Lancet Global Health. 2014; 2(1):e51–7. 10.1016/S2214-109X(13)70118-9 25104636

[4] Picado A, Singh SP, Rijal S et al Longlasting insecticidal nets for prevention of Leishmania donovani infection in India and Nepal: paired cluster randomized trial. 2010;341:c6760 10.1136/bmj.c6760 PMC3011370 21190965

[5] Mondal D, Chowdhury R, Huda MM et al Insecticide-treated bed nets in rural Bangladesh: their potential role in the visceral leishmaniasis elimination programme. Trop Med Int Health. 2010;15(11):1382–9. 10.1111/j.1365-3156.2010.02635.x 20946233

[6] Joshi AB, Das ML, Akhter S, Chowdhury R et al Chemical and environmental vector control as a contribution to the elimination of visceral leishmaniasis on the Indian subcontinent: cluster randomized controlled trials in Bangladesh, India and Nepal. BMC Med. 2009;7:54 10.1186/1741-7015-7-54 19804620 PMC2763005

[7] Messenger LA, Matias A, Manana AN et al Multicentre studies of insecticide-treated durable wall lining in Africa and South-East Asia: entomological efficacy and household acceptability during one year of field use. Malar J. 2012;11:358 10.1186/1475-2875-11-358 23107112 PMC3547731

[8] Saghaei M. Random allocation software: http://mahmoodsaghaei.tripod.com/Softwares/randalloc.html 10.1186/1471-2288-4-26 PMC533876 15535880

[9] Barrera R, Amador M, Clark GG. The use of household bleach to control *Aedes aegypti*. J Am Mosq Control Assoc. 2004; 20(4):444–8. 15669389

[10] Fernández EA, Leontsini E, Sherman C, Chan AS, Reyes CE, Lozano RC, Fuentes BA, Nichter M, Winch PJ. Trial of a community-based intervention to decrease infestation of *Aedes aegypti* mosquitoes in cement washbasins in El Progreso, Honduras. Acta Trop. 1998;70(2):171–83. 9698263 10.1016/s0001-706x(98)00033-3

[11] Sherman C, Fernandez EA, Chan AS, Lozano RC, Leontsini E, Winch PJ. La Untadita: a procedure for maintaining washbasins and drums free of *Aedes aegypti* based on modification of existing practices. Am J Trop Med Hyg. 1998 2;58(2):257–62. 9502612 10.4269/ajtmh.1998.58.257

[12] Kumar V, Kesari SK, Sinha NK, Palit A, Kishore K, Saran R, Kar SK. Field trial of an ecological approach for the control of *Phlebotomus argentipes* using mud and lime plaster. Indian J Med Res. 1995; 101:154–156. 7751045

[13] Kayedi MH, Khamisabadi K, Dehghani N, Haghdoost AA. Entomological evaluation of PermaNet 2.0^®^ and K-O Tab 1-2-3^®^ treated nets in comparison to nets conventionally treated with deltamethrin, after repeated washing. Pathog Glob Health. 2015 6;109(4):196–201. 10.1179/2047773215Y.0000000010 25978624 PMC4530558

[14] Sinton JA. Ibid. Part XIX. The value of female genitalia in the identification of some species. Indian J Med Res. 1927; 44: 263–304.

[15] World Health Organization Communicable Disease Control Prevention and Eradication. Guidelines for Laboratory and Field testing of Mosquito Larvicides. Geneva World Health Organization Pesticide Evaluation Scheme2005. Report No.: WHO/CDS/WHOPES/GCDPP/2005.13.

[16] Chowdhury R, Dotson E, Blackstock AJ, McClintock S, Maheswary NP, Faria S, et al Comparison of insecticidetreated nets and indoor residual spraying to control the vector of visceral leishmaniasis in Mymensingh District, Bangladesh. Am J Trop Med Hyg. 2011;84(5):662–7. 10.4269/ajtmh.2011.10-0682 21540372 PMC3083730

[17] Dinesh DS(1), Das ML, Picado A, Roy L, Rijal S, Singh SP, Das P, Boelaert M, Coosemans M. Insecticide susceptibility of *Phlebotomus argentipes* in visceral leishmaniasis endemic districts in India and Nepal. PLoS Negl Trop Dis. 2010; 4(10):e859 10.1371/journal.pntd.0000859 21049013 PMC2964302

[18] Kroeger A, Villegas E, Morison L: Insecticide impregnated curtains to control domestic transmission of cutaneous leishmaniasis in Venezuela: cluster randomized trial. BMJ. 2002, 325:810–813. 12376442 10.1136/bmj.325.7368.810 PMC128948

[19] Ahmed B, Nabi SG, Rahman M et al Kala-azar (Visceral leishmaniasis) Elimination in Bangladesh: Success and challenges. Curr Trop Med Rep. 2014; 1: 163–169.

[20] KA_Road-Map-NVBDCP. National road map for kala-azar elimination August 2014. Directorate of National Vector Borne Disease Control Programme, Directorate General of Health Services, Ministry of Health and Family Welfare, 22 Sham Nath Marg, Delhi-54 (http://nvbdcp.gov.in/Doc/Road-map-KA_2014.pdf

